# Reviewers

**DOI:** 10.1093/jncics/pkac058

**Published:** 2022-09-16

We wish to thank the following reviewers for their generous assistance in evaluating manuscripts submitted to *JNCI Cancer Spectrum* during the past year.

Thomas Ahearn

Jiyoung Ahn

David Akhavan

Rodrigue Allodji

Arya Amini

Meenakshi Anurag

Julie Armin

Hoda Badr

Christopher Baggett

William Barlow

Christie Befort

Guillaume Beinse

Peter Beitsch

Marianne Berwick

Ana Best

Francois-Clement Bidard

Prunella Blinman

Dejana Braithwaite

Justin Brown

Harry Burke

Mark Buyyounouski

Qiuyin Cai

Gregory Calip

Rikki Cannioto

Ying Cao

David Cescon

Young Chandler

Stephen Chanock

Cheng Cheng

Ting-Yuan David Cheng

Meenalakshmi Chinnam

Hyunsoon Cho

Rebecca Christensen

Niall Corcoran

Giuseppe Curigliano

Suzanne Dahlberg

Monica D'Arcy

Luigi De Petris

Adriano Decarli

Wendy Demark-Wahnefried

Mindy DeRouen

Todd DeWees

Haryana Dhillon

Michaela Dinan

Kevin Dobbin

Edward Ellerbeck

D Gareth Evans

Christine Friedenreich

Byron Gajewski

Seana Gall

Lisa Gallicchio

Phyllis Gimotty

Amanda Graham

Peter Grimison

Petros Grivas

Axel Grothey

Marc Gunter

Michael Gutter

Ann Hamilton

Heather Hampel

Sigurdis Haraldsdottir

Emily Harris

Kathy Helzlsouer

N Lynn Henry

Deirdre Hill

Anette Hjartaker

Carrie Howell

Matthew Hudson

Long Jiang

Corinne Joshu

Aaron Katz

Steven Katz

Kelley Kidwell

Belinda Kiely

Han Kim

Gretchen Kimmick

Anita Kinney

Anne Kirchhoff

Andrea Knezevic

Tzy-Mey Kuo

Allison Kurian

Chris Labaki

Mary Beth Landrum

Yun Li

Jennifer Ligibel

Unhee Lim

Yanhong Liu

Chris Lominska

Wendy London

Hung Luu

Clement Ma

Yong Ma

Jennifer Mack

Kepher Makambi

Sarah Markt

Deborah Mayer

Joseph McGuirk

Tomer Meirson

Kathy Miller

Qianxing Mo

Motomi Mori

Lindsay Morton

Erin Moth

Kent Mouw

Neil Murphy

Sarah Nechuta

Heather Neuman

Matthew Nielsen

Byeongsang Oh

Linda Overholser

Haitao Pan

Electra Paskett

Sebastian Pena

Curtis Pettaway

Liem Phan

Stan Pounds

C.S. Pramesh

Sandi Pruitt

Stephanie Pugh

Kerryn Reding

Melissa Reimers

Anne Reiner

Kimber Richter

Debra Ritzwoller

Kim Robien

Philip Rosenberg

Jonathan Rosenberg

Trevor Royce

Lawrence Rubinstein

Berhe Sahle

Barbara Saltzman

Howard Sandler

Patrick Schnell

Lawrence Schwartz

Xinglei Shen

Qian Shi

David Shibata

Robert Simari

Lena Specht

Susan Steck

Shane Stecklein

Ravid Straussman

Gita Suneja

Ray Tan

Ben Tran

Clare Turnbull

Svitlana Tyekucheva

Janette Vardy

Christine Veenstra

Nicholas Webster

Evertine Wesselink

Mary White

Jan Wohlfahrt

Canhua Xiao

Robert Yarchoan

James Yu

Tian Zhang

